# Lecithin-Stabilized Polymeric Micelles (L_*sb*_PMs) for Delivering Quercetin: Pharmacokinetic Studies and Therapeutic Effects of Quercetin Alone and in Combination with Doxorubicin

**DOI:** 10.1038/s41598-018-36162-0

**Published:** 2018-12-05

**Authors:** Chia-En Chang, Chien-Ming Hsieh, Sheng-Chin Huang, Chia-Yu Su, Ming-Thau Sheu, Hsiu-O. Ho

**Affiliations:** 1 School of Pharmacy, College of Pharmacy, Taipei Medical University, Taipei, Taiwan; 2 Clinical Research Center and Traditional Herbal Medicine Research Center, Taipei Medical University Hospital, Taipei, Taiwan

## Abstract

In this study, lecithin-stabilized polymeric micelles (L_*sb*_PMs) were prepared to load quercetin (QUE) in order to improve its bioavailability and increase its antitumor activity. Its combination with doxorubicin (DOX) to minimize DOX-mediated cardiac toxicity and increase the antitumor activity of QUE-loaded L_*sb*_PMs was also examined. L_*sb*_PMs were prepared following a previously reported procedure. Results demonstrated that optimal QUE-loaded L_*sb*_PMs contained quercetin, D-α-tocopheryl polyethylene glycol succinate, and lecithin at a weight ratio of 6:40:80. Drug-release studies showed that QUE released from L_*sb*_PMs followed a controlled release pattern. A cytotoxicity assay revealed that QUE-loaded L_*sb*_PMs had significant anticancer activities against MCF-7, SKBR-3, and MDA-MB-231 human breast cancer cells and CT26 mouse colon cancer cells. In animal studies, intravenous administration of QUE-loaded L_*sb*_PMs resulted in efficient growth inhibition of CT26 colon cancer cells in a Balb/c mice model. In a pharmacokinetics study compared to free QUE, intravenous and oral administration of QUE-loaded L_*sb*_PMs was found to have significantly increased the relative bioavailability to 158% and 360%, respectively, and the absolute bioavailability to 5.13%. The effect of QUE-loaded L_*sb*_PMs in combination with DOX resulted in efficient growth inhibition of CT26 colon cancer cells and reduced cardiac toxicity in the Balb/c mice model.

## Introduction

Dietary phytochemicals have been found to be efficacious against cancer in cancer epidemiology and experimental. Daily consumption is a new approach to prevent cancer. In a review, Lee *et al*.^1^ described that dietary phytochemicals could inhibit tumor progression and block tumor initiation. Non-toxic therapeutic drugs or dietary phytochemicals provided an effective method to cancer therapy strategies^2^. These could induce good results containing cell-cycle arrest, autophagy, differentiation, and apoptosis^1^. Thus, phytochemicals as dietary constituents are being examined for their cancer preventive and treatment potentials^3^. Since quercetin (QUE) is a major constituent of various dietary products, its cancer prevention and treatment potentials have been extensively explored. Several previous reports presented an excellent overview of research related to therapeutic applications of QUE in cancer prevention and treatment^4–8^.

Although QUE possesses great medicinal value, its use as a therapeutic agent is hampered by its poor oral bioavailability. Being ascribed to class IV of the Biopharmaceutical Classification System (BCS), the low bioavailability of QUE is a consequence of its lipophilic character of low solubility in water (0.17~7.7 μg/mL), artificial gastric juice (5.5 μg/mL), and artificial intestinal juice (28.9 μg/mL)^9^, as well as its pre-systemic metabolism (lower permeability) by both intestinal efflux pumps (e.g., P-glycoprotein and MRP2) and cytochrome P450 (CYP3A)^10,11^, all of which are abundantly present in the epithelium of the gastrointestinal tract^12^. Based on those results, it is recognized that in order to promote the oral absorption of QUE and its oral bioavailability, the aqueous solubility of QUE must be improved, and pre-systemic metabolism and the efflux effect have to be prevented. QUE-loaded nanocarrier systems, including polymer nanoparticles^12^, microemulsions and self-emulsifying systems^13,14^, liposomes^15–18^, micelles^19–22^, and solid lipid particulates^23^, have been evaluated as potential ways to resolve these two main hurdles in recent years. The clinical usefulness of QUE for cancer chemoprevention and treatment either intravenously or orally delivered by various nanocarrier systems was also demonstrated^24–26^.

In our previous report, self-assembling lecithin-based mixed polymeric micelles (_*sa*_LMPMs) were successfully developed as a drug delivery system for QUE to improve its solubility and bioavailability^21^. However, the stability of QUE-loaded _*sa*_LMPMs needed to be improved to be applicable for clinical use. Because of that, lecithin-stabilized polymeric micelles (L_*sb*_PMs) recently successfully developed for delivering docetaxel in our lab were utilized to accomplish this purpose^27^. The design rationale for L_*sb*_PMs was that the lipid layer can be fused onto PMs by ultrasonication to form a supporting lipid layer, which possesses better stability and encapsulates more hydrophobic drug than micelles leading to enhancement of the therapeutic efficacy.

Doxorubicin (DOX) is one of the most potent anticancer drugs; however, its use is limited by the risk of severe cardiotoxicity. This is generally thought to be caused by free radicals generated during redox cycling of DOX and/or the cardiotoxic action of doxorubicinol, a C13-dihydrometabolite of DOX^28^. Therefore, pharmacological agents which are able to suppress the formation of both doxorubicinol and reactive oxygen species merit intense investigation^29^. QUE is considered a strong antioxidant due to its ability to scavenge free radicals and bind transition metal ions. These properties of QUE allow it to inhibit lipid peroxidation^30^. Therefore, a combination of QUE and DOX was expected to potentiate the antitumor efficacy of DOX against cancer cells and simultaneously mitigate DOX-generated cardiotoxicity.

Herein, we established a process for preparing L_*sb*_PMs with PMs stabilized with lipids by subjecting them to ultrasonication during hydration of the thin film composed of an amphiphilic polymer and QUE. We examined formulation and processing factors that affected preparation of the L_*sb*_PMs in an effort to find an optimal formulation, and subsequently characterized the particle size, physical stability, drug-release prolife, *in vitro* cell toxicity, *in vivo* pharmacokinetics (PKs), biodistribution, and tumor-inhibitory efficacy in a xenograft mice model of optimized L_*sb*_PMs. The therapeutic efficacy and cardiotoxicity of a therapeutic combination of QUE-loaded L_*sb*_PMs and DOX were also evaluated.

## Results

### Optimization of QUE-loaded L_*sb*_PMs

In a preliminary study, D-α-Tocopheryl polyethylene glycol succinate (TPGS) was found to be a suitable amphiphilic polymer for loading QUE. Optimization of QUE-loaded L_*sb*_PMs based on TPGS is given in Table 1. It reveals that a QUE:TPGS:lecithin ratio of 6:40:80 of L_*sb*_PMs was optimal, with a particle size of 92.2 ± 0.35 nm, a polydispersity index (PI) of 0.468 ± 0.016, an encapsulation efficiency (EE) % of 98.88%, and a drug loading (DL) % of 4.72%. It further demonstrated the longest stability of 5 days after reconstitution among all formulations examined. It was selected for the following study.

**Table 1:** Optimization of quercetin (QUE)-loaded lecithin-stabilized polymeric micelles (L_*sb*_PMs).

Quercetin (mg)	TPGS (mg)	Lecithin (mg)	size (nm) (PI) mean ± SD	EE (%)	DL (%)	Stability
		20	>3000			PPT
	30	40	>3000			PPT
		60	>3000			PPT
		80	>3000			PPT
		20	>3000			PPT
	40	40	>3000			<15 min
		60	>3000			<15 min
		80	92.2 ± 0.35 (0.468 ± 0.016)	98.88	4.72	<5 days
	50	50	>3000			<1 h
		60	97.6 ± 3.07 (0.653 ± 0.078)	95.25	5.03	<3 days
		70	84.3 ± 2.12 (0.453 ± 0.321)	93.36	4.84	<3 days
6	60	50	97.6 ± 3.07 (0.653 ± 0.078)	93.37	5.34	<3 days
		60	83.3 ± 5.07 (0.638 ± 0.023)	93.23	4.89	<3 days
	70	30	83.4 ± 0.63 (0.837 ± 0.031)	86.81	4.82	<2 days
		40	83.1 ± 2.26 (0.503 ± 0.056)	91.47	5.24	<3 days
		50	74.3 ± 0.26 (0.534 ± 0.039)	94.26	4.38	<3 days
	80	30	83.2 ± 0.54 (0.637 ± 0.038)	87.55	4.02	<1 day
		40	73.4 ± 0.34v (0.643 ± 0.023)	92.56	4.81	<2 days
	90	20	>3000			<1 h
		30	85.5 ± 2.33 (0.510 ± 0.090)	95.68	5.01	<3 days

Abbreviations: PI, polydispersity index; PPT, precipitation; EE, encapsulation efficiency; DL, drug loading.

### Physical Characterizations of Optimal QUE-loaded L_*sb*_PMs

The morphology of QUE-loaded L_*sb*_PMs was observed using TEM imaging. TEM images depicted in Fig. S1 reveal that lecithin-stabilized micelles were spherical and uniform and had mean particle sizes of ~90 nm.

The functional stability of QUE-loaded L_*sb*_PMs was evaluated based on changes in the particle size and PI value in various media exposed to 37 °C. After dilution with PBS and FBS, QUE-loaded L_*sb*_PMs were observed to be quite stable, as they maintained a similar size of nearly 90 nm and had a similar PI as the initial materials in both PBS and FBS media for at least 5 days (data not shown).

**Figure 1:**
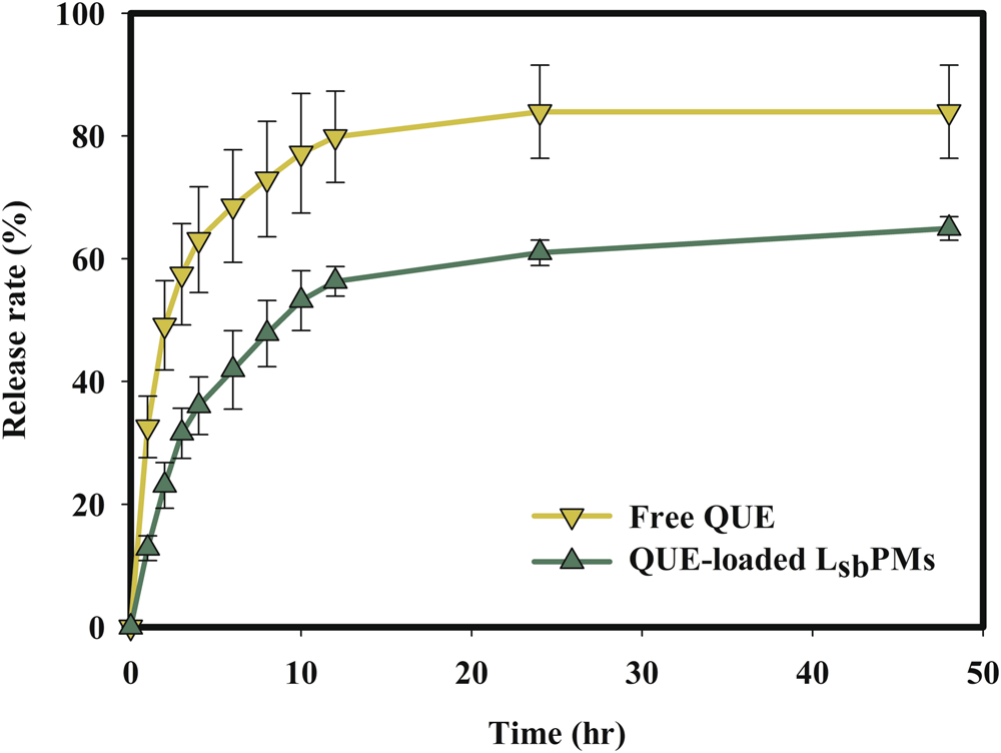
Drug release profile of free quercetin (QUE) and QUE-loaded lecithin-stabilized polymeric micelles (L_*sb*_PMs) in pH 7.4 phosphate buffer with the addition of 0.5% Tween 80. Each point is shown as the mean ± SD (*n* = 3).

To evaluate the release kinetics of QUE from QUE-loaded L_*sb*_PMs *in vitro*, a dialysis method was utilized in 10 mM PBS (pH 7.4) that contained 0.5% Tween 80 at 37 °C. As shown in Fig. 1, compared to free QUE, the release of QUE from QUE-loaded L_*sb*_PMs was slightly slower and occurred to a lesser extent. In the case of free QUE, more than 80% of QUE was released during the first 10 h, whereas only 60% of the QUE amount was released from QUE-loaded L_*sb*_PMs in that time.

### Cell Viability Assays

The *in vitro* anticancer effect of QUE-loaded L_*sb*_PMs compared to free QUE was assessed by an MTT assay. As shown in Table 2, values of the half maximal inhibitory concentration (IC_50_) of QUE-loaded L_*sb*_PMs at 24 h against MCF-7, SKBR-3, MDA-MB-231, and CT26 cells were all lower than those for free QUE. It was more obvious that free QUE was ineffective at inhibiting the growth of MCF-7 and SKBR-3 cells. Table 2 also illustrates that placebo L_*sb*_PMs (without QUE) were observed to have some extent of inhibition on the growth of the four cell lines examined. But IC_50_ values of the four cell lines were higher than those for QUE-loaded L_*sb*_PMs incubated with different doses of QUE-loaded L_*sb*_PMs.

**Table 2:** Values of the 50% inhibitory concentration (IC_50_; µg/ml) of quercetin (QUE)-loaded lecithin-stabilized polymeric micelle (L_*sb*_PM) formulations against MCF-7, SKBR-3, MDA-MB-231, and CT26 cancer cell lines.

Cancer cell	MCF-7	SKBR-3	MDA-MB-231	CT26
Formulation				
QUE-loaded L_*sb*_PMs	1.41	4.63	3.18	1.86
Placebo L_*sb*_PMs	6.41	7.73	5.82	2.48
Free QUE	>30	>30	28.4	4.81

### *In Vivo* PK Study of Intravenous and Oral Administration

Figure S2 shows the plasma concentration of QUE versus time for SD rats after they had intravenously received 50 mg/kg (Fig. S2A) or were orally administered 100 mg/kg (Fig. S2B) of QUE-loaded L_*sb*_PMs and free QUE. PK parameters for both routes are listed in Table 3. Figure S2A illustrates similar plasma profiles for the IV administration of QUE-loaded L_*sb*_PMs and free QUE, but with a higher AUC_0−∞_ and a longer T_1/2_ for the former. As shown in Table 3, the AUC value of QUE-loaded L_*sb*_PMs was calculated to be 1.58-times higher than that of free QUE, while a longer T_1/2_ of 10.84 ± 5.24 h (vs. 7.31 ± 6.73 h). Figure S2B shows that the plasma profile of QUE after administration of a single 100-mg/kg oral dose of QUE-loaded L_*sb*_PMs obviously differed from that for free QUE. The PK parameters shown in Table 3 demonstrate that a higher Cmax value of 1.52 ± 1.31 µg/mL observed for QUE-loaded L_*sb*_PMs than that of 0.53 ± 0.22 µg/mL for free QUE. Similarly, the AUC_0−∞_ of 0.90 ± 0.26 h·µg/mL for QUE-loaded L_*sb*_PMs was greater than that of 0.25 ± 0.12 h·µg/mL for free QUE. The absolute oral bioavailabilities (BAs) of QUE-loaded L_*sb*_PMs and free QUE with respect to IV administration of free QUE were calculated to be 5.13% and 1.42%, respectively. The relative oral BA of QUE-loaded L_*sb*_PMs with respect to free QUE was found to be 3.6-fold higher.

**Table 3:** Pharmacokinetic parameters of quercetin (QUE) after intravenous and oral administration of QUE-loaded lecithin-stabilized polymeric micelles (L_*sb*_PMs) and free QUE to SD rats.

	Free QUE (IV)	QUE-loaded L_*sb*_PMs (IV)	Free QUE (Oral)	QUE-loaded L_*sb*_PMs (Oral)
Dose (mg/kg)	50	50	100	100
T_max_ (h)	N/A	N/A	0.25	0.25
T_1/2_ (h)	7.31 ± 6.73	10.84 ± 5.24	N/A	N/A
C_max_ (µg/ml)	49.65 ± 15.76	87.99 ± 28.64	0.53 ± 0.22	1.52 ± 1.31
AUC_0-inf_ (h·µg/ml)	17.51 ± 6.15	27.74 ± 8.82	0.25 ± 0.12	0.90 ± 0.26
V (l/kg)	24.32 ± 15.28	26.23 ± 14.8	N/A	19.76 ± 14.05
CL (l/min/kg)	3.12 ± 1.44	1.78 ± 0.44	N/A	102.29 ± 42.80
BA (%) (IV-IV)	100	158		
BA (%) (IV-Oral)			1.42	5.13
BA (%) (Oral-Oral)			100	360

Data are presented as the mean ± SD deviation (*n* = 5).

Abbreviations: T_max_, the time to reach C_max_; T_1/2_, half-life of the drug; C_max_, maximum plasma drug concentration; AUC_0–inf,_ area under the receiver operating curve at 0 to infinity; V, volume of distribution; CL, clearance; BA, bioavailability.

### Anticancer Effects of the IV Administration of QUE-loaded L_*sb*_PMs and Free QUE in the CT26 Tumor Model

**Figure 2:**
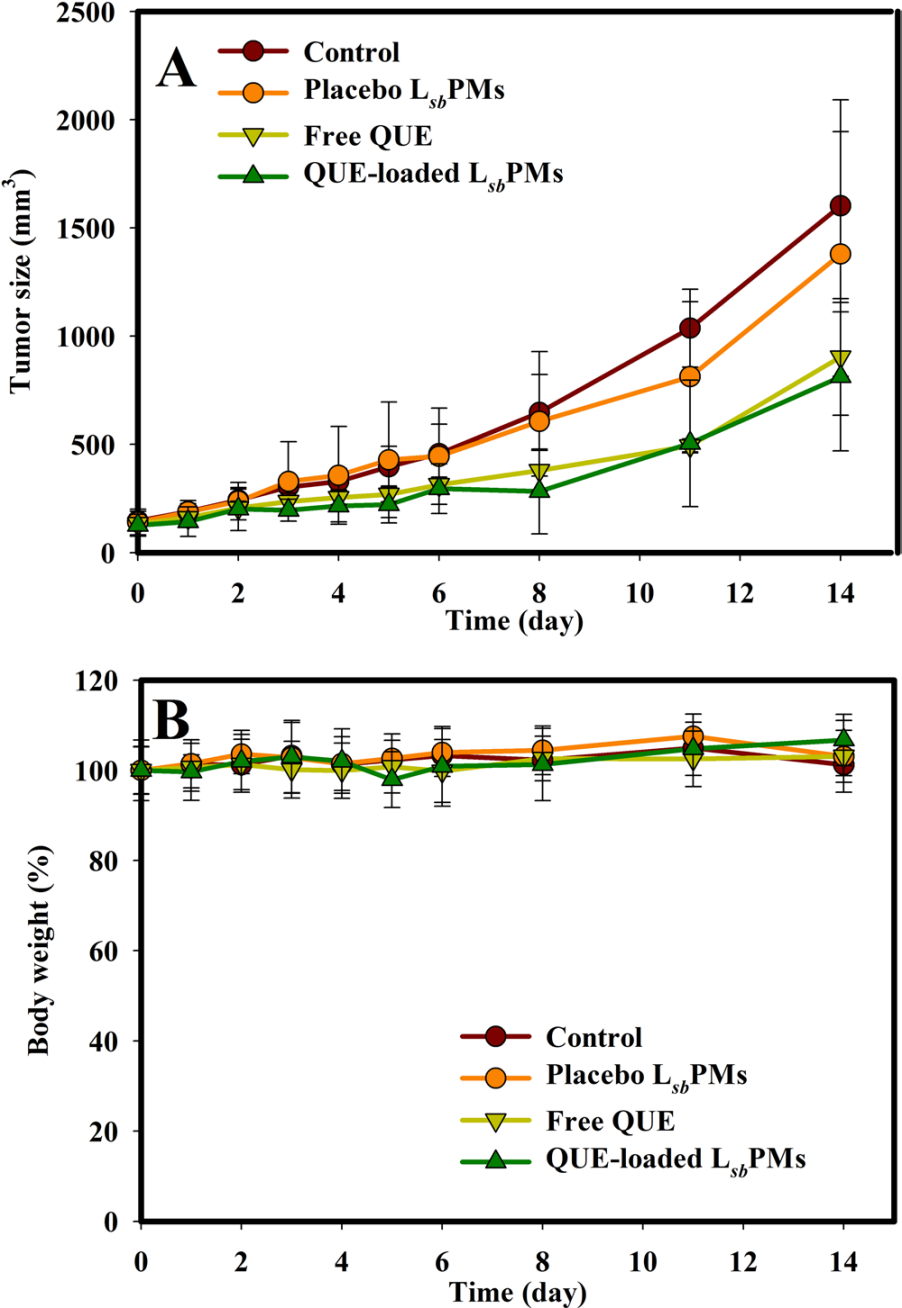
Changes in (**A**) the tumor volume and (**B**) body weight after an intravenous injection of PBS, placebo lecithin-stabilized polymeric micelles (L_*sb*_PMs), free QUE, and QUE-loaded L_*sb*_PMs Q1d*7 at 50 mg/kg. Each point is shown as the mean ± SD (*n* = 5).

Since lower IC_50_ values were observed for QUE-loaded L_*sb*_PMs and free QUE in the CT26 tumor cell line, it was selected to be the tumor model for evaluating the antitumor efficacy. Figure 2 displays the tumor growth profile of CT26 cells (Fig. 2A) and body weight changes (Fig. 2B) after IV administration of the PBS control, placebo L_*sb*_PMs, free QUE, and QUE-loaded L_*sb*_PMs. Results demonstrated that the order of tumor volumes was ranked as QUE-loaded L_*sb*_PMs (50.76%) ≤free QUE (56.40%) <placebo L_*sb*_PMs (86.07%) <PBS control (100%). Compared to the PBS control, QUE-loaded L_*sb*_PMs and free QUE showed significantly greater extents of tumor inhibition with a slightly higher extent of tumor inhibition for the former, which correlated with a low IC_50_ of 1.86 µg/mL for the former versus 4.81 µg/mL for the latter observed in terms of cell viability. On the other hand, the extent of tumor growth inhibition for placebo L_*sb*_PMs was lower, which was inconsistent with a lower IC_50_ value of 2.48 µg/mL observed in the cell viability study. This indicates that the cell cytotoxicity of placebo L_*sb*_PMs with an IC_50_ value of 2.48 µg/mL did not correlate well with the *in vivo* tumor inhibition effects, and the tumor growth inhibitory effects of QUE-loaded L_*sb*_PMs were mainly attributed to the QUE load.

### Therapeutic and Cardioprotective Effects of the Co-administration of QUE-loaded L_*sb*_PMs with DOX in the CT26 Tumor Model

**Figure 3:**
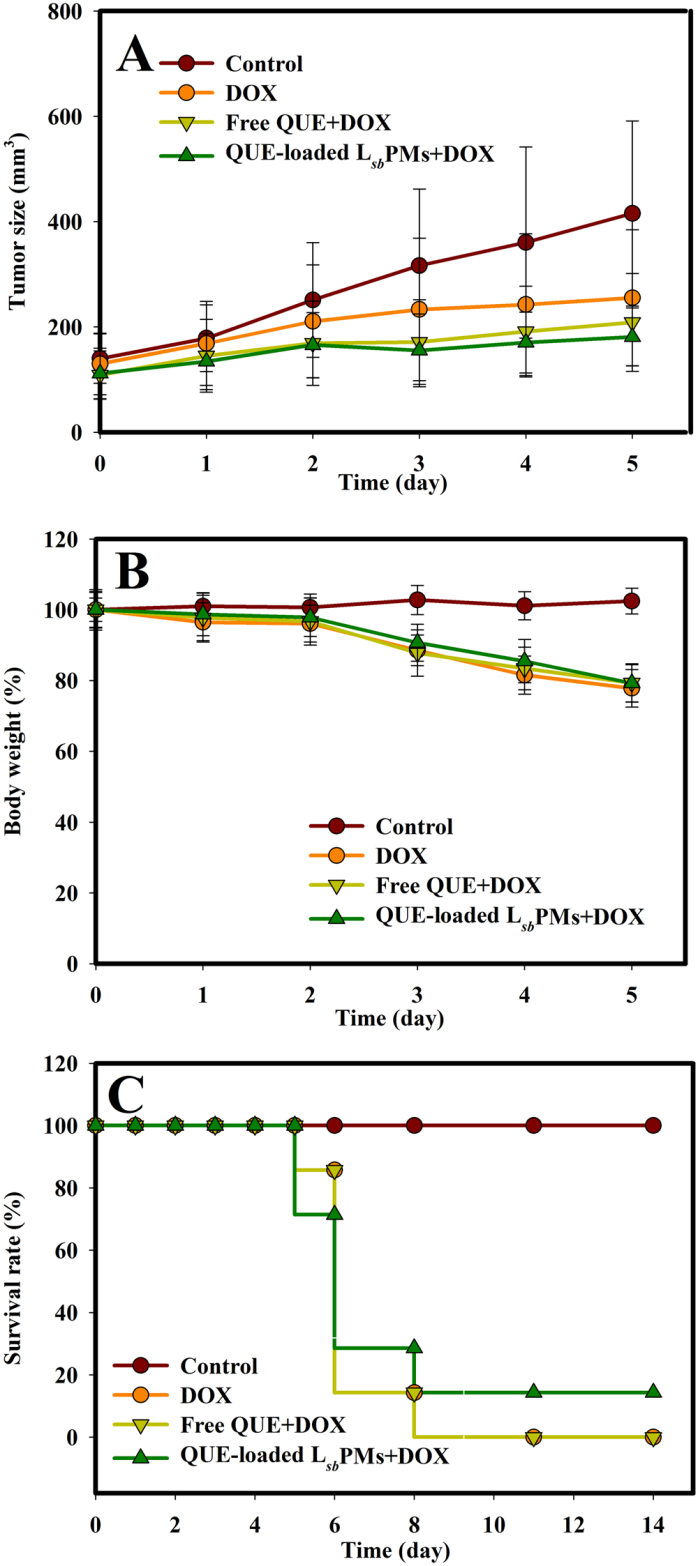
Changes in (**A**) the tumor volume, (**B**) body weight, and (**C**) survival rate after an intravenous injection of PBS, doxorubicin (DOX), free quercetin (QUE) plus DOX, and QUE-loaded lecithin-stabilized polymeric micelles (L_*sb*_PMs) plus DOX at Q1d*5, QUE at 50 mg/kg, and DOX at 4 mg/kg. Each point is shown as the mean ± SD (*n* = 5).

**Figure 4:**
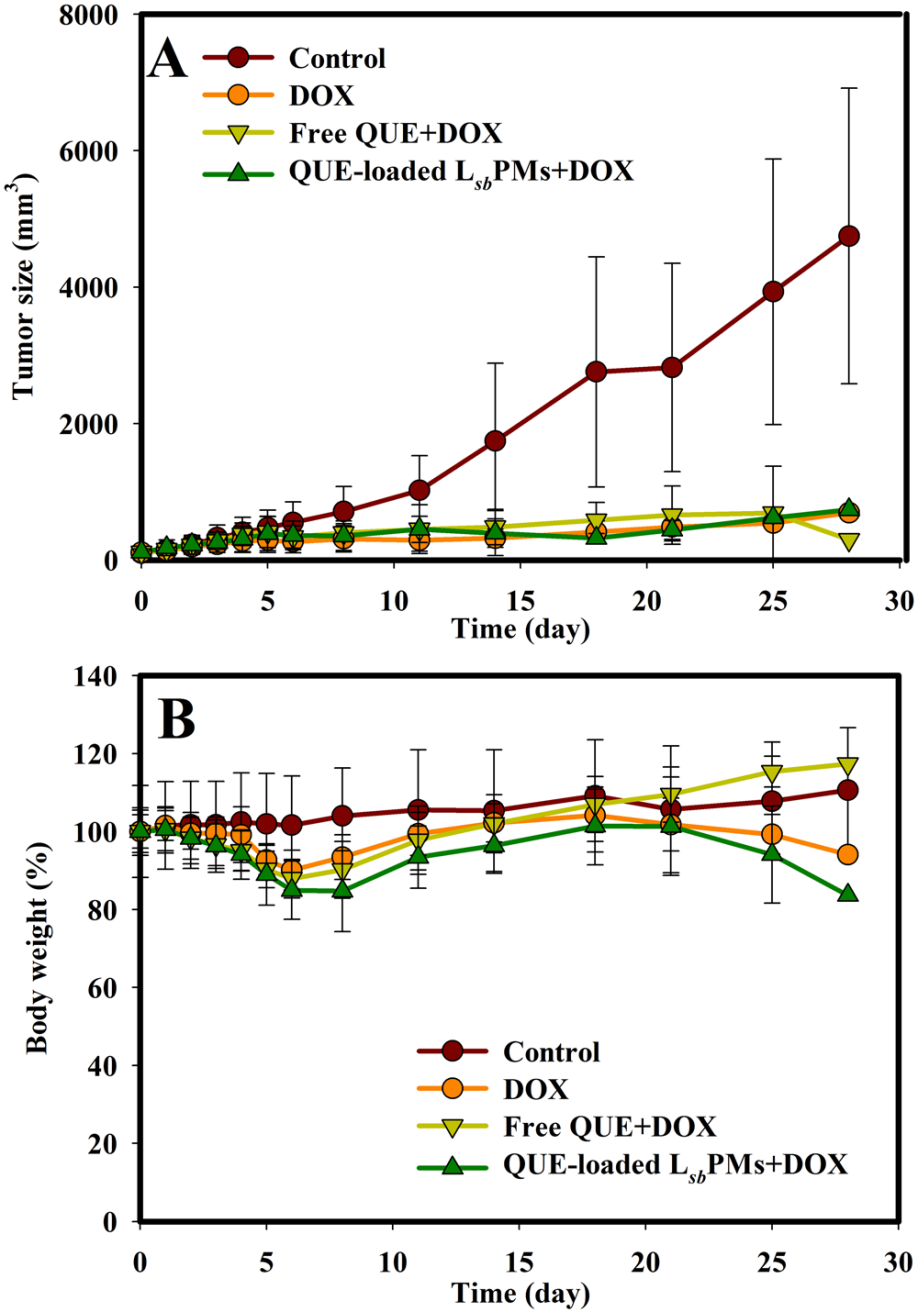
Changes in (**A**) the tumor volume and (**B**) body weight after an intravenous injection of PBS, doxorubicin (DOX), free quercetin (QUE) plus DOX, and QUE-loaded lecithin-stabilized polymeric micelles (L_*sb*_PMs) plus DOX at Q1d*5, QUE at 50 mg/kg, and DOX at 2 mg/kg. Each point is shown as the mean ± SD (*n* = 5).

Therapeutic and cardioprotective activities of QUE-loaded L_*sb*_PMs and free QUE combined with DOX via intravenous administration were evaluated at a fixed QUE dose of 50 mg/kg with two DOX doses (4 and 2 mg/kg), and results are shown in Figs 3 and 4 for DOX doses of 4 and 2 mg/kg, respectively. Results in Fig. 3A demonstrate that the ranking order of tumor volumes after treatment was as follows: PBS control group (100%) >DOX group (54.21%) >free QUE + DOX (50.12%) >QUE-loaded L_*sb*_PMs + DOX (43.37%). Except for the PBS control group, body weights for all other groups were observed to have a >20% decline by the fifth day as shown in Fig. 3B. The survival profile shown in Fig. 3C further illustrates that mice began to die on the sixth day in all groups with the exception of the PBS control group. Figure 3C nevertheless displays that a slightly better survival rate profile was shown for QUE-loaded L_*sb*_PMs + DOX. This overall indicates that QUE-loaded L_*sb*_PMs could improve the therapeutic efficacy of DOX at a 4-mg/kg dose and enhance the survival rate.

Results in Fig. 4A illustrate that the ranking order of tumor volumes after administration of treatments was as follows: PBS control (100%) >DOX (14.55%) >free QUE + DOX (14.53%) >QUE-loaded L_*sb*_PMs + DOX (13.03%), indicating the nearly complete inhibition of tumor growth by all treatments, with QUE-loaded L_*sb*_PMs showing a slight improvement in the therapeutic efficacy. Body weights for all groups were observed to have had no more than a 20% change at the end of the 28-day observation period as shown in Fig. 4B. Dissimilar to treatments at a DOX dose of 4 mg/kg, there were no rat deaths during this time period. It was concluded that QUE-loaded L_*sb*_PMs had at least slightly improved the therapeutic efficacy of DOX at a 2-mg/kg DOX dose, while showing significantly lower DOX-induced systemic toxicity and causing no rat deaths.

Cardioprotective activities of QUE were evaluated based on histopathological examinations of heart tissue samples collected after each treatment. Morphological alterations of lesions presented as the histopathology incidence grade microscopically observed for heart tissue samples are shown in Table 4 for the group treated with QUE-loaded L_*sb*_PMs in combination with doses of 4 and 2 mg/kg DOX. Results of the histopathology incidence grade shown in Table 4 illustrate an insignificant difference in morphological alterations of lesions, including myocyte vacuolar degeneration, myocyte degeneration/necrosis, and inflammatory cell infiltration with collagen disposition for heart tissue samples collected on the final day of treatment. However, it was observed for heart tissue samples collected on day 8 after treatment that the degree of lesions in myocyte vacuolar degeneration for the treatment group with free DOX only was graded as moderate (histopathological grade of 3, 26~50%), while a minimal degree of lesions (histopathological grade of 1, <1%) was graded for the two other treatment groups combining DOX at a 4-mg/kg dose with either QUE-loaded L_*sb*_PMs or free QUE. It was concluded that even given at a 4-mg/kg DOX dose, cardioprotective activities of QUE in QUE-loaded L_*sb*_PMs and free QUE were observed as a result of QUE in both forms being distributed or taken up at similar extents into the heart.

**Table 4:** Histopathology incidence after injection of doxorubicin (DOX), quercetin (QUE)-loaded lecithin-stabilized polymeric micelles (L_*sb*_PMs) plus DOX, and free QUE plus DOX at Q1d*5, QUE at 50 mg/kg, and DOX at 4 or 2 mg/kg, respectively.

DOX dose (mg/kg)	4						2					
Time	Day 5			Day 8			Day 5			Day 28		
Formulation	I	II	III	I	II	III	I	II	III	I	II	III
Vacuolar degeneration, myocytes	0	1	0	3	1	1	0	1	2	2	1	1
Degeneration/necrosis, myocytes	0	0	1	1	1	1	1	2	3	4	2	2
Inflammatory cell infiltration with collagen deposition	0	0	0	1	0	0	1	2	2	2	2	1

Note: The degree of lesions was graded from 1 to 5 depending on the severity: 0 = normal; 1 = minimal (<1%); 2 = slight (1~25%); 3 = moderate (26~50%); 4 = moderately severe (51~75%); 5 = severe/high (76~100%).

I = free DOX; II = Free QUE + DOX; III = QUE-loaded L_*sb*_PMs + DOX.

Table 4 illustrates the histopathology incidence grade of morphological alterations of lesions including myocyte vacuolar degeneration, myocyte degeneration/necrosis, and inflammatory cell infiltration with collagen deposition for heart tissue samples collected at 3- or 4-day intervals until day 28 and on the final day of treatment. Compared to the histopathology incidence grade of lesions for heart tissue samples collected on day 28, it was observed that the degree of lesions of myocyte vacuolar degeneration, myocyte degeneration/necrosis, and inflammatory cell infiltration with collagen deposition for the treatment group with free DOX only was graded as slight (histopathological grade of 2, 1~25%), moderate (histopathological grade of 4, 26~50%), and slight (histopathological grade of 2, 1~25%), respectively, while those for treatment groups of free QUE and QUE-loaded L_*sb*_PMs were minimal (histopathological grade of 1, <1%) and minimal (histopathological grade of 1, <1%), slight (histopathological grade of 2, 1~25%) and slight (histopathological grade of 2, 1~25%), and slight (histopathological grade of 2, 1~25%) and minimal (histopathological grade of 1, <1%), respectively.

## Discussion

In a previous report^21^, an optimal level of _*sa*_LMPMs for loading QUE was revealed to be QUE:lecithin:P123 in a weight ratio of 3:1:20, with a particle size of 78.7 nm, an EE% of 88.07%, and a DL% of 11.01%. However, their physical stability after reconstitution was not good enough for clinical applications. Therefore, L_*sb*_PMs as reported^31^ were utilized for loading QUE in an attempt to improve their physical characteristics. The optimal QUE-loaded L_*sb*_PMs contained quercetin, D-α-tocopheryl polyethylene glycol succinate, and lecithin at a weight ratio of 6:40:80. TPGS is a water-soluble derivative of natural vitamin E, which is formed by esterification of vitamin E succinate with PEG. As such, it has advantages of PEG and vitamin E in applications of various nanocarriers for drug delivery, including an extended half-life of the drug in plasma and enhanced cellular uptake of the drug. TPGS has an amphiphilic structure of a lipophilic alkyl tail and a hydrophilic polar head with a hydrophilic/lipophilic balance (HLB) value of 13.2^32^ and a relatively low critical micelle concentration (CMC) of 0.02% w/w, which make it an ideal molecular biomaterial for developing various drug delivery systems, including prodrugs, micelles, liposomes, and nanoparticles. It allows the realization of sustained, controlled, and targeted drug delivery, overcomes multidrug resistance (MDR), and promotes oral drug delivery as an inhibitor of P-glycoprotein (P-gp)^33^. The release of QUE from QUE-loaded L_*sb*_PMs was slightly slower and occurred to a lesser extent. This demonstrates that QUE entrapped in L_*sb*_PMs was able to be sustainably released, thus preventing its clearance by the systemic circulation. A cytotoxicity assay revealed that QUE-loaded L_*sb*_PMs had significant anticancer activities. This indicates that encapsulation of QUE by L_*sb*_PMs enhanced the cytotoxic activity of QUE. Further, TPGS promoted the uptake of loaded QUE or inhibition of P-gp efflux, especially in MCF-7 and SKBR-3 cells, leading to lower IC_50_ values for all four cell lines. In pharmacokinetic study, the AUC value and T_1/2_ of QUE-loaded L_*sb*_PMs were higher than free QUE, indicated that indicated that the clearance of QUE encapsulated in L_*sb*_PMs from systemic circulation was slower, making QUE more bioavailable. The relative oral BA of QUE-loaded L_*sb*_PMs was found to be higher than free QUE. This indicates that encapsulation of QUE in L_*sb*_PMs was able to enhance the oral absorption of QUE, leading to significant increases in the relative BA. Also mechanisms involving by TPGS inhibiting P-gp activity, TPGS can be attributed for this significant bioavailability enhancement^33^. The combination of either free QUE or QUE-loaded L_*sb*_PMs was able to reduce DOX-induced cardiotoxicity with slightly more-efficacious long-term protection for the combination of QUE delivered by QUE-loaded L_*sb*_PMs. This might be attributed to a sustained plasma concentration profile after IV administration of QUE-loaded L_*sb*_PMs.

## Conclusions

L_*sb*_PMs were prepared following the same process conditions with optimal examination to select TPGS as a suitable amphiphilic substance to combine with lecithin resulting in formation of QUE-loaded L_*sb*_PMs. QUE-loaded L_*sb*_PMs showed lower IC_50_ values against MCF-7, SKBR-3, and MDA-MB-231 human breast cancer cells and CT26 mouse colon cancer cells. In animal studies, the intravenous administration of QUE-loaded L_*sb*_PMs resulted in efficient growth inhibition of CT26 colon cancer cells in a Balb/c mice model. In a pharmacokinetics study, compared to free QUE, intravenous and oral administration of QUE-loaded L_*sb*_PMs was able to enhance the extents of relative bioavailability to 158% and 360%, respectively, and the absolute bioavailability to 5.13%. The effect of DOX in combination with QUE-loaded L_*sb*_PMs resulted in efficient growth inhibition of CT26 colon cancer cells with reduced DOX-induced cardiotoxicity. It was concluded that QUE-loaded L_*sb*_PM formulations could potentially be an acceptable nanocarrier with greater stability for delivering hydrophobic phytochemicals that are expected to enhance the antitumor efficacy of chemotherapy and reduce systemic toxicities.

## Experimental Section

### Materials

QUE and acetic acid were obtained from Sigma-Aldrich (St. Louis, MO, USA). Methanol and acetonitrile were purchased from JT Baker (Center Valley, PA, USA). Fetal bovine serum (FBS) was purchased from Biowest (Nuaillé, France). D-α-Tocopheryl polyethylene glycol succinate (TPGS) was purchased from BASF (Ludwigshafen, Germany). Lecithin S100 (soya lecithin) was purchased from Lipoid (Ludwigshafen, Germany). Matrigel was procured from Becton Drive (Franklin Lakes, NJ, USA). Tween^®^ 80 was purchased from Merck (Billerica, MA, USA). All reagents used for the high-performance liquid chromatographic (HPLC) analysis, including acetonitrile and methanol, were HPLC grade, and other reagents were analytical grade.

### Preparation of QUE-loaded L_*sb*_PMs

QUE-loaded L_*sb*_PMs were prepared by a thin-film hydration method. Briefly, QUE and TPGS were dissolved in methanol at a predetermined ratio. The mixture was subsequently evaporated using a rotary evaporator at 50 °C under reduced pressure to obtain a thin film, and the film was further dried overnight at room temperature to remove any residuals. A lecithin nanosuspension was prepared as follows: 1000 mg soybean lecithin (S-100) was suspended in 25 mL of deionized water and then subjected to ultrasonication (VCX 750, at a frequency of 20 kHz, Sonics and Materials, Newtown, CT, USA) to form a lecithin nanosuspension to hydrate the polymeric thin film. Then 1 mL of the lecithin nanosuspension containing various amounts of lecithin was used to hydrate the thin film containing QUE and a hydrophilic polymer, and the mixture was again ultrasonicated at full power for at least 5 min while maintaining a constant temperature to obtain the nanocarrier solution. After formation of the micelles, unincorporated QUE aggregates were removed by passing the solution through a 0.22-µm filter (Millipore, Billerica, MA, USA). Characteristics of QUE-loaded L_*sb*_PMs in the filtrate were evaluated, such as the average particle size and size distribution, encapsulation efficiency (EE), and drug loading (DL).

### Physical Characterization of QUE-loaded L_*sb*_PMs

The mean particle size and size distribution of QUE-loaded L_*sb*_PMs were measured using an N5 submicron particle size analyzer (Beckman Coulter, Brea, CA, USA) at room temperature. All measurements were performed in triplicate. The morphology was observed using transmission electron microscopy (TEM; Hitachi H-700, Tokyo, Japan).

### Quantification of QUE in QUE-loaded L_*sb*_PMs

The encapsulated amount of QUE in QUE-loaded L_*sb*_PMs was determined by an HPLC method. (for details see ‘Experimental Methods’ section in Supplementary material).

### Functional Stability of QUE-loaded L_*sb*_PMs

To evaluate the stability of QUE-loaded L_*sb*_PMs, they were diluted in phosphate-buffered saline (PBS) and fetal bovine serum (FBS) and then stored at 37 °C. Then the particle size and size distribution (polydispersity index; PI) were determined every day for 3 days.

### *In Vitro* Release Studies

*In vitro* release kinetics of QUE-loaded L_*sb*_PMs were assessed using a dialysis bag method (for details see ‘Experimental Methods’section in Supplementary material).

### Cell Viability Assays

The extents of cytotoxicity of QUE-loaded L_*sb*_PMs and free QUE to SKBR-3, MCF-7, and MDA-MB-231 breast cancer cells and CT26 colon cancer cells were evaluated using an MTT assay. Cells were seeded at a density of 5 × 10^4^ cells/well in 24-well plates. After 24 h of treatment at 37 °C with 5% CO_2_, cell survival was measured using a tetrazolium salt MTT assay. One hundred microliters of MTT (6 mg/mL) was added to each well and incubated for 3 h. The medium was then removed, and 200 μL of dimethyl sulfoxide was added to dissolve any purple formazan crystals formed. Cell viability was measured with a spectrophotometer (Bio-Tek, Winooski, VT, USA); the absorbance was set to 520 nm.

### *In Vivo* PK Study of Intravenous and Oral Administration

The protocol of this study was approved by the Institutional Animal Care and Use Committee of Taipei Medical University (approval No. LAC-101-0063), and all experiments were performed under approved animal care guidelines by Taipei Medical University (Taipei, Taiwan). Male Sprague-Dawley (SD) rats aged 8~10 weeks were purchased from BioLASCO Taiwan (Taipei, Taiwan) and used to investigate the PK profile of QUE-loaded L_*sb*_PMs and free QUE (QUE dissolved with EtoH:Tween^®^ 80 = 1:1 to 40 mg/mL and then diluted with DDW to 6 mg/mL). Rats (five rats per group) were administered a single 50-mg/kg dose through a tail vein injection. Blood samples were collected in heparinized tubes from the jugular vein at 0.083, 0.25, 0.5, 1, 2, 3, 8, 12, 24, and 48 h after administration. In addition, rats (five rats per group) were randomly assigned to two groups and orally administered a single 100-mg/kg dose of QUE-loaded L_*sb*_PMs or free QUE. Blood samples were collected in heparinized tubes from the jugular vein at 0.25, 0.5, 1, 1.5, 2, 3, 4, 6, 8, 12, 24, and 48 h after oral administration. All blood samples were immediately centrifuged at 3000 rpm for 15 min to obtain plasma, which was stored at −30 °C before the HPLC analysis. PK parameters were calculated for each group using the mean and standard deviation (SD) from individual rats and estimated using a noncompartmental analysis. The maximum plasma concentration (Cmax) and the time to reach Cmax (Tmax) were directly obtained from the observed concentration-time curve data. The terminal elimination rate constant (Ke) was estimated according to the slope of the log-linear phase of declining plasma concentrations versus time graph. The half-life (T_1/2_) was calculated using the following equation: T_1/2_ = ln(2/Ke). The area under the concentration time curve (AUC_0→last_) was calculated using the trapezoidal method. Summing the AUC_0→last_ and the concentration at the last measured point divided by Ke yielded AUC_0→inf_. Clearance (CL) was calculated by dividing the dose by AUC_0→inf_, and the volume of the distribution (V) was obtained by dividing CL by the Ke.

### The Anticancer Effects of the IV Administration of QUE-loaded L_*sb*_PMs and Free QUE in the CT26 Tumor Model

The CT26 tumor cell line was used to evaluate the antitumor efficacy. CT26 cell suspensions (100 µL) containing 5 × 10^4^ cells in PBS/matrix gel were subcutaneously implanted into the right thighs of mice. CT26 tumor-bearing mice with 150-mm^3^ tumor volumes were randomly assigned to four experimental groups (*n* = 5 mice/group): one negative control group (PBS), one placebo L_*sb*_PM group, one free QUE group, and one QUE-loaded L_*sb*_PM group. Each animal was injected at a dose of 50 mg/kg once a day for 7 days. Body weights and tumor volumes were measured twice weekly, and the tumor volume (V) was calculated by the formula:

$${\rm{V}}=[{\rm{length}}\times {({\rm{width}})}^{2}]/2.$$

### Therapeutic and Cardioprotective Effects of the Co-administration of QUE-loaded L_*sb*_PMs with DOX in the CT26 Tumor Model

CT26 cell suspensions (100 µL) containing 5 × 10^4^ cells in PBS/matrix gel were subcutaneously implanted into the right thighs of mice. CT26 tumor-bearing mice with 150-mm^3^ tumor volumes were randomly assigned to four experimental groups (*n* = 5 mice/group): one PBS control group, one DOX group, one free QUE + DOX group, and one QUE-loaded L_*sb*_PMs + DOX group. Each animal was injected at a dose of 50 mg/kg of QUE plus 2 or 4 mg/kg of DOX once a day for 5 days. Body weights and tumor volumes were measured every day (in the 4-mg/kg DOX group) or twice weekly (in the 2-mg/kg DOX group), and the tumor volume (V) was calculated by the formula:

$${\rm{V}}=[{\rm{length}}\times {({\rm{width}})}^{2}]/2.$$

The survival rate was also evaluated and defined as the percentage of mice in the treatment group still alive for a certain period of time after initiation of treatment.

Cardiotoxicity effects after the two experimental designs of giving either a 2- or 4-mg/kg dose of DOX with a fixed QUE dose in QUE-loaded L_*sb*_PMs were evaluated based on heart tissue specimens isolated from one mouse of each treatment group in one experiment design sampling at the end of drug administration and at a predetermined time. Heart tissue samples were fixed in 10% neutral buffered formalin for 48 h at room temperature, dehydrated in increasing concentrations of ethanol, cleared with xylene, and embedded in paraffin. Approximately 4~5-μm-thick sections were prepared from a tissue paraffin block and stained with hematoxylin and eosin (H&E). Tissue samples on the slide were examined for any structural changes under light microscopy to assess myocardium injury by an animal pathology expert at the National Applied Research Laboratories (Taipei, Taiwan). The severity of lesions was graded according to methods described by Shackelford *et al*.^34^. The extent of the lesions was graded histopathologically from 0 to 5 depending on the severity (0 = normal, 1 = minimal (<1%); 2 = slight (1~25%); 3 = moderate (26~50%); 4 = moderately severe (51~75%); and 5 = severe/high (76~100%). Lesions related to acute DOX-mediated cardiotoxicity were characterized by disorganized myofibrils, hyalinization of myofibrils, increased vacuolization, and swelling of organelles, while those related to chronic DOX-mediated cardiotoxicity were scattered vacuolated myocytes.

### Statistical Analysis

Statistical analysis of all results was performed by Student’s *t*-test assuming unequal variance. Two-tailed *p* values of <0.05 were regarded as statistically significant differences. Tabulated data are presented as the mean ± SD.

## Electronic supplementary material

Supplementary Dataset 1
