# Supplementary material for: Lecithin-Stabilized Polymeric Micelles (LsbPMs) for Delivering Quercetin: Pharmacokinetic Studies and Therapeutic Effects of Quercetin Alone and in Combination with Doxorubicin
Source: Sci Rep. 2018 Dec 5;8:17640. doi: 10.1038/s41598-018-36162-0 (PMC6281656; doi:10.1038/s41598-018-36162-0)
Supplement: Supplementary file 1 — Supplementary Dataset 1 [file 41598_2018_36162_MOESM1_ESM.pdf]

Supplementary Information:

Title of manuscript:

Lecithin-Stabilized Polymeric Micelles (L_{sb}PMs) for Delivering Quercetin: Pharmacokinetic Studies and Therapeutic Effects of Quercetin Alone and in Combination with Doxorubicin

Authors:

Chia-En Chang[1*] , Chien-Ming Hsieh[1*], Sheng-Chin Huang[1], Chia-Yu Su[1], Ming-Thau Sheu[1,2#] and Hsiu-O Ho[1#]

*The first two authors contributed equally to this work.
[#]The Corresponding two authors equally to this work.
Corresponding author: Hsiu-O Ho
Address: 250 Wuxing St. Taipei 11031, Taiwan
Telephone: 886-2-27361661 ext. 6126
Fax: 886-2-23771942
E-mail: hsiuoho@tmu.edu.tw

Supplementary information includes:

Supplemental information includes supplemental methods; two figures can be found with this article online.

Experimental Methods

Quantification of QUE in QUE-loaded L_{sb}PMs

The encapsulated amount of QUE in QUE-loaded L_{sb}PMs was determined by an HPLC method. First, a supernatant solution of QUE-loaded L_{sb}PMs was filtered through 0.22-µm filter paper. The solution (20 µl) was diluted with 980 µl of methanol:H_2O (6:4), and then determined through an HPLC method using an Inertsil® ODS-3 column (C18, 6 µm, 4.6 x 150 mm; GL Sciences, Torrance, CA, USA). The column oven temperature was maintained at 40 °C. The mobile phase was methanol:0.025 M phosphate buffer (60:40, v/v). The flow rate was 1 mL/min. The detection wavelength was set to 375 nm, and the sample injection volume was 20 µL. The QUE concentration was determined from the calibration curve, and the EE percentage (EE%) and DL percentage (DL%) were respectively calculated according to equations 1 and 2:

$$EE\% = WM/WI \times 100 \text{ and} \quad (1)$$

$$DL\% = WM/(WP + WM) \times 100; \quad (2)$$

where WM is the weight of the drugs in the micelles, WI is the weight of the initial feeding drug, and WP is the weight of the initial feeding polymers.

In Vitro Release Studies

After being diluted to a concentration of 1 mg/mL, 1 ml of diluted free QUE (QUE dissolved in EtOH:Tween®80 at 1:1 to 40 mg/mL and then diluted with double-distilled water (DDW) to 6 mg/mL) and QUE-loaded L_{sb}PMs were placed in a dialysis bag (MWCO = 6000 Da) and immersed in 20 mL of 10 mM PBS (pH 7.4) containing 0.5% Tween 80 at 37 °C with shaking at 100 rpm. The incubation medium was replaced with fresh medium at predetermined intervals. The QUE concentration was determined by the HPLC method described above.

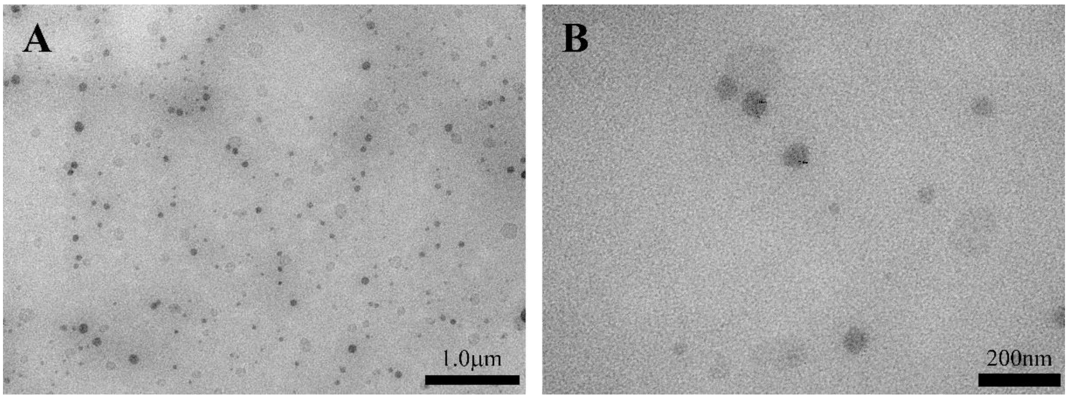

Figure S1 TEM photographs of quercetin (QUE)-loaded lecithin-stabilized polymeric micelles (L_{sb}PMs) (QUE:TPGS:lecithin = 6:40:80, w/w/w) at (A) 12,000x and (B) 50,000x magnification.

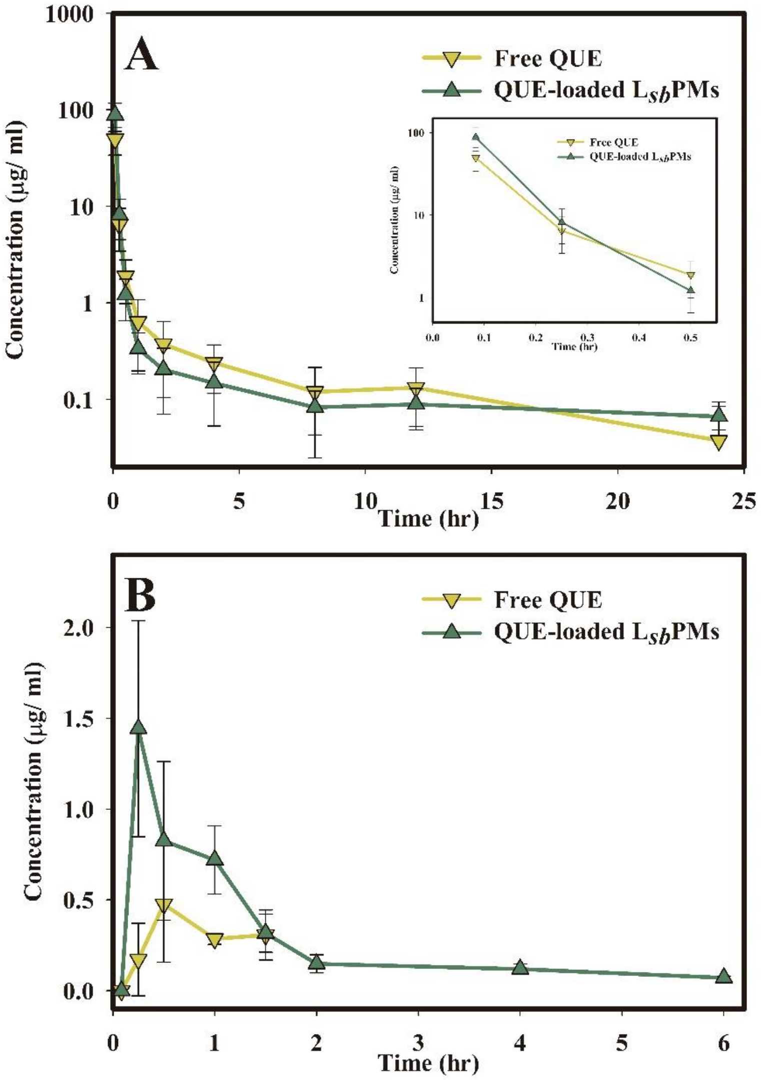

Figure S2 Plasma concentration-time curves of quercetin (QUE) after (A) intravenous (50 mg/kg) and (B) oral (100 mg/kg) administration of QUE-loaded lecithin-stabilized polymeric micelles (L_{sb}PMs) and free QUE to rats. Each point is shown as the mean ± SD (*n*=5).
